# A Macroscopic Reaction: Direct Covalent Bond Formation between Materials Using a Suzuki-Miyaura Cross-Coupling Reaction

**DOI:** 10.1038/srep06348

**Published:** 2014-09-18

**Authors:** Tomoko Sekine, Takahiro Kakuta, Takashi Nakamura, Yuichiro Kobayashi, Yoshinori Takashima, Akira Harada

**Affiliations:** 1 Department of Macromolecular Science, Graduate School of Science, Osaka University, Toyonaka, Osaka 560-0043, Japan

## Abstract

Cross-coupling reactions are important to form C–C covalent bonds using metal catalysts. Although many different cross-coupling reactions have been developed and applied to synthesize complex molecules or polymers (macromolecules), if cross-coupling reactions are realized in the macroscopic real world, the scope of materials should be dramatically broadened. Here, Suzuki-Miyaura coupling reactions are realized between macroscopic objects. When acrylamide gel modified with an iodophenyl group (I-gel) reacts with a gel possessing a phenylboronic group (PB-gel) using a palladium catalyst, the gels bond to form a single object. This concept can also be adapted for bonding between soft and hard materials. I-gel or PB-gel selectively bonds to the glass substrates whose surfaces are modified with an electrophile or nucleophile, respectively.

Adhesives with various functional properties are distributed worldwide^1^,^2^. Modern chemistry has developed synthetic adhesives that bond materials. Although bonding materials with similar qualities is relatively easy, bonding different materials (e.g., a glass-polymer film and a metal-polymer film) is more difficult and requires pretreatment using a suitable method. Actually, to bond a hard material and a polymer film, a primer layer, which serves as an anchor, is coated on the hard material prior to bonding with the polymer films. If accomplished a direct bonding between two materials without a primer reagents, that method will create a new paradigm in adhesion science.

Two methods may realize direct bonding between materials: a method with non-covalent bond formation and one using covalent bond formation. Adhesion between the same materials through non-covalent bonds reversibly forms bonds^3^,^4^,^5^,^6^,^7^,^8^, and bonding materials via this method does not require primer reagents. Non-covalent bonds formed via hydrogen bonds^9^, π–π stacking interactions^10^,^11^,^12^, metal–ligand coordination bonds^13^, ionic interactions^14^, and hydrophobic interactions^15^,^16^,^17^ act as glue on the molecular level and are useful for self-healable polymeric materials utilizing spontaneous reformation of chemical bonds. Studies using non-covalent bonds have focused on reversible bond formation in the same material and not on materials with different hardness and organic/inorganic qualities. To form robust bonds between materials, non-covalent bond are disadvantageous compared to covalent bonds. Although coupling reactions between polymers in solutions are widely studied^18^,^19^,^20^, studies on bonding between different materials via covalent bond formation are relatively rare.

On the basis of these concepts, we study the adhesion between supramolecular hydrogels possessing host and guest polymers via non-covalent bonds^21^. Pieces of host and guest gels, which are chemically crosslinked acrylamide-based gels with either cyclodextrins (CDs) or small hydrocarbon group guest moieties, adhere to one another via mutual molecular recognition of CDs and the hydrocarbon groups on the surfaces^22^,^23^. These studies have achieved the adhesion between independent soft materials through non-covalent interactions.

We expect that if suitable organic electrophiles and nucleophiles are modified on two independent materials, direct bonding between materials via a cross-coupling reaction would be observed without glue in the presence of a catalyst. Here, we chose the palladium-catalyzed Suzuki-Miyaura cross-coupling reaction^24^ to covalently bond soft and hard materials. A phenylboronic acid (PB) group as a nucleophile or an iodophenyl (I) group as an electrophile is modified on a scaffold material, poly(acrylamide) gel. Bondings between two hydrogel materials and between a hydrogel (organic compound) and a hard material (inorganic compound) are investigated on the macroscopic scale using a cross-coupling reaction at their interface.

## Results

### Preparation of PB-gel and I-gel

**Figure 1:**
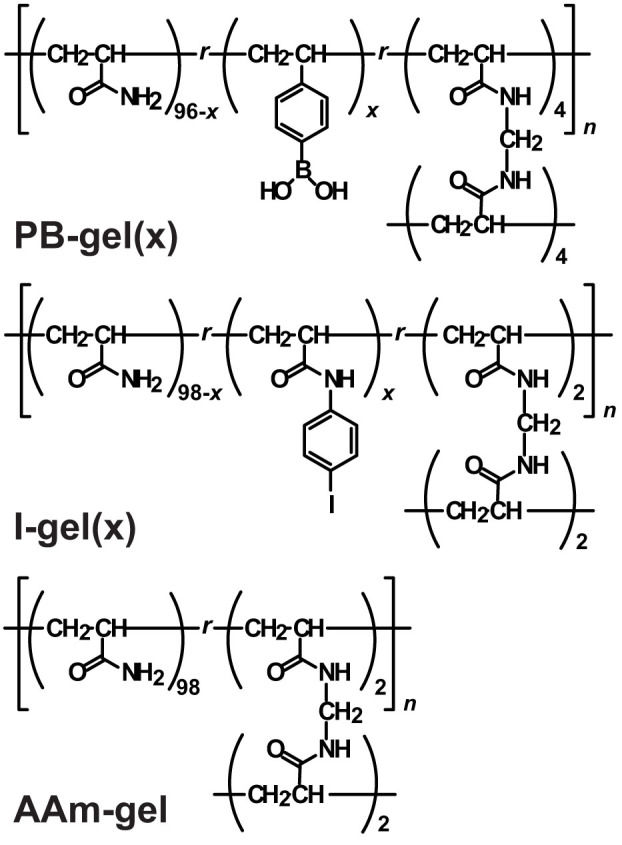
Chemical structures of PB-gel(*x*), I-gel(*x*), and AAm-gel. *x* denotes the mol% of functional groups (PB group or I group). *N,N′*-methylenebisacrylamide (MBAAm) unit is standardized to 4 mol% in PB-gel(*x*), 2 mol% in I-gel(*x*), and 2 mol% in AAm-gel.

To achieve adhesion between soft materials through Suzuki-Miyaura coupling reactions, we selected a PB group as a nucleophile and an I group as an electrophile. Figure 1 depicts the chemical structure between a hydrogel with the PB group (PB-gel(*x*)) and that with the I group (I-gel(*x*)), in which *x* represents the mol% of the functional group (PB or I group). In this study, acrylamide-based gels bearing a PB or I group are employed due to the lack of interaction between polyacrylamides. The amounts of *N,N′*-methylenebisacrylamide (MBAAm) units as a chemical crosslinker are 4 mol% in PB-gel(*x*) and 2 mol% in I-gel(*x*).

PB-gel(*x*) was obtained by copolymerization of acrylamide (AAm), MBAAm, and 4-vinylphenyl boronic acid monomers in dimethyl sulfoxide (DMSO) using 2,2′-azobis(isobutyronitrile) (AIBN) as an initiator (Scheme S1, Table S1). Prior to the preparation of I-gel(*x*), a scaffold gel (AA-gel: poly(acrylic acid)-*r*-poly(acrylamide) gel) was initially prepared by homogeneous radical copolymerization of AAm, MBAAm, and acrylic acid (AA) monomers. The condensation reaction of 4-iodoaniline with the AA-gel gave I-gel(*x*) (Scheme S2 and Table S2). As a reference gel, AAm-gel with 2 mol% of MBAAm, which lacks a nucleophile or electrophile, was prepared. Each gel was purified by washing with DMSO to remove the unreacted compounds. In the final hydrogels, the solvent was replaced with water.

Characterization by solid-state ^1^H field gradient magic angle spinning (FGMAS) nuclear magnetic resonance (NMR) and Fourier transform infrared (FT-IR) spectroscopies confirmed the chemical structures and content of each monomeric unit of the PB-gel(*x*) and I-gel(*x*) (Supplementary Figures S1–S2 and Tables S3–S4). For example, the ^1^H FGMAS NMR spectra of PB-gel(5) and I-gel(5) demonstrate that the 5.1 mol% of PB group and the 4.7 mol% of I group are introduced into each gel, indicating that the functional groups are introduced into the gels according to the feed ratio. Tables S3 and S4 summarize the mol% of the functional groups in the gels. A PB-gel(5) or I-gel(5) measuring 5 × 4 × 3 mm^3^ contains about 0.006 mmol of functional groups.

### Adhesion between the PB-gel and I-gel

**Figure 2:**
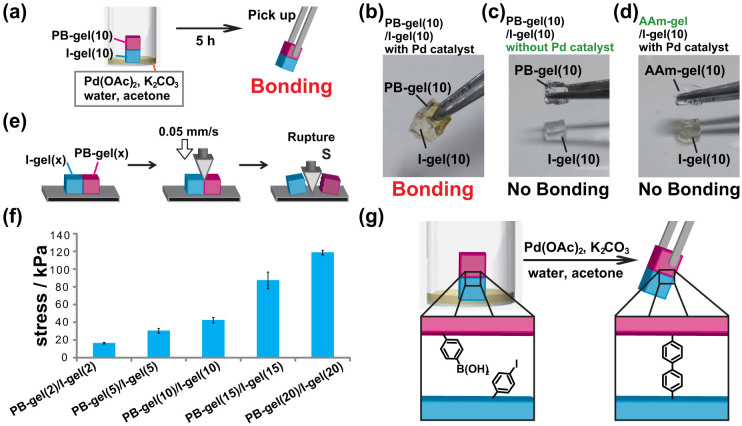
Coupling reaction on the contact interface using a Pd(OAc)_2_ catalyst. (a) Adhesion experimental procedure of PB-gel with I-gel. Cuboid-shaped PB-gel(10) (size: 5 × 4 × 3 mm^3^) and I-gel(10) are immersed in an aqueous solution of K_2_CO_3_ (0.72 M, 3 mL, depth: 2 mm) and acetone solution of palladium (II) acetate (Pd(OAc)2) (0.01 M, 30 μL) for 5 hours. Results of adhesion experiments between (b) PB-gel(10)/I-gel(10) with the Pd(OAc)_2_ catalyst, (c) PB-gel(10)/I-gel(10) without a catalyst, and (d) AAm-gel/I-gel(10) with a catalyst. (e) Experimental procedure of the wedged-shaped strain compression test. (f) Stress values between PB-gel(*x*)/I-gel(*x*) where the error bars denote the standard deviation of three samples. (g) Proposed mechanism of the biphenyl bond formation at the gel–gel interface.

We carried out the coupling reaction at a contact interface between a PB-gel and an I-gel. Figure 2a shows the experimental procedure of the interfacial coupling reaction. I-gel(10) and PB-gel(10) were cut into cuboid-shapes (size: 5 × 4 × 3 mm^3^) and stacked vertically. The stacked gels were then immersed in an aqueous solution of K_2_CO_3_ (0.72 M, 3 mL, depth: 2 mm). Next an acetone solution containing palladium(II) acetate (Pd(OAc)_2_) (0.01 M, 30 μL) was added.

After standing for 5 hours, the PB-gel(10) strongly bonds to the I-gel(10) (Figure 2b, Movie S1). The PB-gel and I-gel do not bond when immersed in a solution without the Pd(OAc)_2_, even if the two pieces remain in contact for a long time (over 72 hours) (Figure 2c). Additionally, in the presence of Pd(OAc)_2_, the AAm-gel without a functional reactive group for Suzuki-Miyaura coupling, does not bond to I-gel(10) (Figure 2d). These results indicate that the interfacial covalent bonding via a Suzuki-Miyaura coupling reaction plays an important role to bond PB-gel and I-gel.

The bonding strengths of the two gels were evaluated by a wedged-shaped strain compression test. Figure 2e shows the experimental procedure of the rupture experiment using the wedge-shaped plunger (*θ* = 45°). Figures 2f and S3 show the rupture strength of bound PB-gel(*x*)/I-gel(*x*) as functions of the PB and I unit ratio, respectively. The rupture strength of PB-gel(*x*)/I-gel(*x*) treated in the presence of the catalyst proportionally increases as the content of the PB group or I group in the hydrogels increases. In contrast, the combination of PB-gel(*x*)/I-gel(*x*) without a catalyst does not have a measurable rupture strength. Because the PB group and I group are homogeneously dispersed on the surface and inside the gels, the functional groups are introduced into the gels according to the feed ratio.

Additionally, the rupture strength in PB-gel(10)/I-gel(10) over time was investigated. The rupture strength increases with reaction time and saturated within 15 hours (Figure S4), indicating that the Suzuki-Miyaura coupling reaction between the PB-gel and I-gel forms a biphenyl group proceeds (Figure 2g). Consequently, the number of covalent bonds formed via the coupling reaction can regulate the bonding strength (i.e., changing the molar content of the PB or I groups in the gels or by adjusting the reaction time can alter the bonding strength).

### Coupling reactions on model polymers

**Figure 3:**
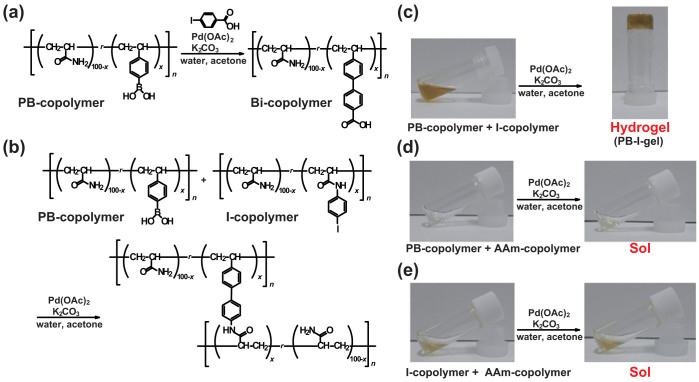
Coupling reactions using model polymers. (a) Reaction of PB-copolymer and 4-iodoaniline, and (b) that of PB-copolymer and I-copolymer. (c) Model coupling reaction of PB-copolymer to I-copolymer to give a hydrogel. (d) Control experiments of (c) using PB-copolymer and AAm-copolymer, and (e) I-copolymer and AAm-copolymer (e). *x* denotes the mol% of the functional groups (PB group or I group).

To verify the Suzuki-Miyaura coupling reaction on the polymer side chain of the gel scaffold, we investigated model polymer systems without chemical crosslinkers (Figure 3). A PB-copolymer, which is an analogue of PB-gel, was prepared by radical copolymerization of 4-vinylphenyl boronic acid with the AAm monomer (Scheme S3). The coupling reaction of 4-iodobenzoic acid with the PB-copolymer using Pd(OAc)_2_ at 25°C in water gave a copolymer with a biphenyl carboxylic acid moiety in high yield (Fig. 3a and Scheme S4). ^1^H NMR measurements of the product polymer display signals assigned to the biphenyl unit (Fig. S5), indicating that the PB groups in the PB-copolymer have an adequate reactivity for the coupling reaction with the Pd catalyst.

A PB-copolymer and an I-copolymer were allowed to react in water in the presence of a Pd(OAc)_2_ catalyst (Figure 3b and Scheme S6). The I-copolymer was obtained by the amide condensation reaction between the AA-copolymer and 4-iodoaniline (Scheme S5). After the reaction in the presence of the Pd catalyst, a mixture of the PB-copolymer and the I-copolymer gives a hydrogel (PB-I-gel) chemically crosslinked due to the formation of biphenyl units (Figure 3c). On the other hand, a mixture of the PB-copolymer and the AAm-polymer or a mixture of the I-copolymer and the AAm-polymer does not provide a hydrogel (Figures 3d and 3e). The dynamic viscoelastic measurements of the PB-I-gel show that values of the storage elastic modulus, *G′*, do not relax (*G′* > *G″*, (*G″*: loss elastic modulus)) in the frequency range of 1 rad·s^−1^ (Table S5). These results indicate that the functional groups of the polymer side chain have a sufficient reactivity toward macromolecules in the coupling reaction using a Pd catalyst.

### Adhesion between hydrogels and glass substrates

**Figure 4:**
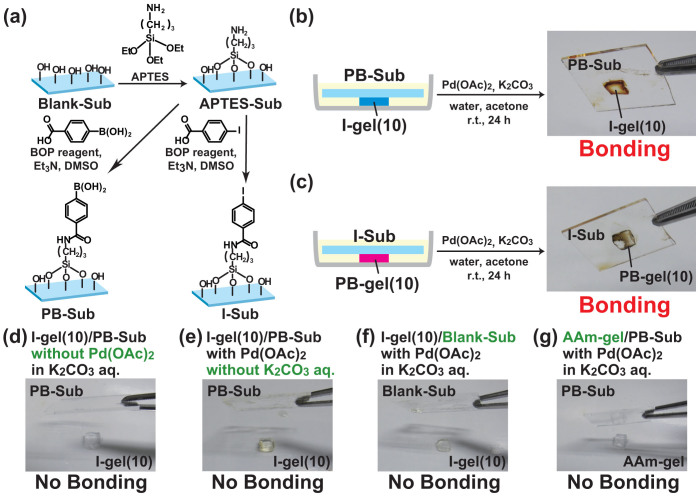
Bonding between hydrogels and glass substrates through the Suzuki-Miyaura cross-coupling reaction. (a) Preparation of PB-Sub and I-Sub. (b) Bonding experimental procedure of I-gel(10) with PB-Sub: PB-Sub is put on I-gel(10) and they are subsequently immersed in an aqueous solution of K_2_CO_3_ (0.72 M, 3 mL, depth: 2 mm). Then an acetone solution of Pd(OAc)_2_ (0.01 M, 30 μL) is added. The gels are then allowed to sit for 24 hours. (c) Bonding experimental procedure of PB-gel with I-Sub. (d)–(g) Results of the bonding experiments between (d) I-gel(10)/PB-Sub without Pd(OAc)_2_ in K_2_CO_3_ aq., (e) I-gel(10)/PB-Sub with Pd(OAc)_2_ without K_2_CO_3_, (f) I-gel(10)/Blank-Sub with Pd(OAc)_2_ in K_2_CO_3_ aq., and (g) AAm-gel/PB-Sub with Pd(OAc)_2_ in K_2_CO_3_ aq. Combinations in (d)–(g) do not bond.

The above bonding experiments were carried out between hydrogels (i.e., soft materials). As a more scientifically challenging target, we also investigated bonding between soft and hard materials. To immobilize electrophilic and nucleophilic molecules on hard materials, a glass substrate treated with (3-aminopropyl)triethoxysilane (APTES) was functionalized with PB or I derivatives, respectively. The APTES glass substrate was immersed into a DMSO solution of the corresonding carboxylic acid or amide condensing reagents and subsequently washed with toluene to remove unreacted reagents. Hereafter, these substrates are called PB-Sub and I-Sub, respectively (Figure 4a and Scheme S7).

The contact angle measurements (Figure S6), Fourier transform infrared (FT-IR) spectrometry (Figure S7), and X-ray photoelectron spectroscopy (XPS) measurements (Figure S8) characterized the substituent groups on PB-Sub and I-Sub. Contact angle measurements confirm the wettability of the modified surfaces (Figure S6). For a water droplet, Glass-Sub shows a contact angle of 36°. After treatment with O_3_/UV, the contact angle of Blank-Sub changed to 3.4°, which is attributed to the hydrophilic surface. For APTES-Sub, which is hydrophobic (84°), the contact angle changes to 42° and 70° after reactions with PB and I derivatives, respectively. These results indicate the functional groups are modified. XPS spectra of both Subs show the corresponding peaks of nitrogen (N 1s) at 398 eV, indicating immobilization of APTES. PB-Sub shows a new peak at 189 eV, which corresponds to boron (B 1s). Similarly, I-Sub exhibits peaks assigned to iodine at 630 eV (I 3d_3/2_) and 618 eV (I 3d_5/2_). These results indicate that the functional groups are successfully immobilized on glass substrates.

The bonding experiments between I-gel(10) and PB-Sub were performed using the procedure shown in Fig. 4b. PB-Sub was placed on I-gel(10), and the two gels were immersed in an aqueous solution of K_2_CO_3_ (0.72 M, 3 mL, depth: 2 mm). Then an acetone solution of Pd(OAc)_2_ (0.01 M, 30 μL) was added. PB-Sub and I-gel(10) were then allowed to sit at room temperature for 24 hours. The I-gel(10) strongly bonds to PB-Sub (Figure 4b). Similarly, PB-gel(10) bonds to I-Sub under the same conditions (Figure 4c). However, the I-gel(10) and PB-Sub do not bond without Pd(OAc)_2_ or K_2_CO_3_ (Figures 4d and e). Using gels or substrates without suitable electrophilic and nucleophilic groups (*i.e.*, I-gel(10)/Blank-Sub and AAm-gel/PB-Sub gels) causes the substrates not to bond (Figures 4f and g). These results indicate that a suitable combination between the PB group and the I group is important to create bonding between hydrogels and glass substrates via a Suzuki-Miyaura cross-coupling reaction.

**Figure 5:**
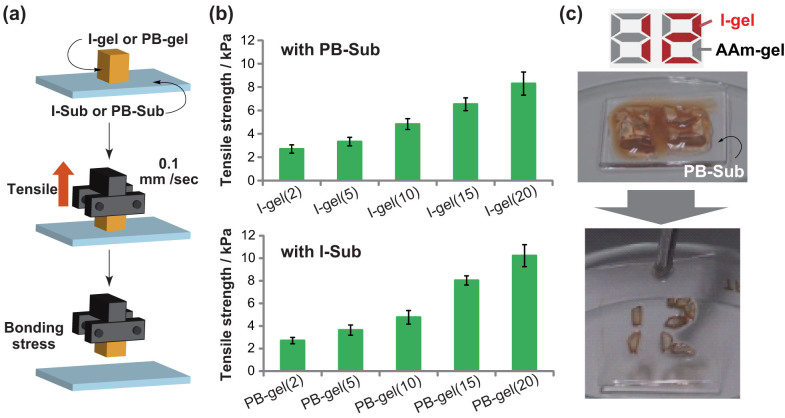
(a) Procedure to measure the tensile strength of the hydrogels and glass substrates. (b) Tensile strengths of I-gel(*x*)/PB-Sub and PB-gel(*x*)/I-Sub as a function of the PB or I unit ratio in the hydrogels. Error bars are standard deviations from three samples. (c) Selective bonding of I-gels on PB-Sub with a Pd catalyst, which depicts the number “12”. After washing with water, AAm-gels disassociate from PB-Sub, while I-gels stick to the surface of PB-Sub.

Next, the bonding strengths between the functionalized gels and glass substrates were evaluated by tensile tests via the following procedure (Fig. 5a). (1) PB-Sub was placed on I-gel(10), immersed into an aqueous solution of K_2_CO_3_ (0.72 M, 3 mL, depth: 2 mm), and an acetone solution of Pd(OAc)_2_ (0.01 M, 30 μL) was added. PB-Sub and I-gel(10) were then allowed to sit for 24 hours. (2) The PB-Sub was immobilized on the sample stage and the I-gel was held by a jig clip. (3) The jig clip was lifted at a rate of 0.1 mm/sec. The tensile strength (*S*), which is the value at which the gel detaches from the glass substrate, was then measured.

Figure 5b shows the tensile strengths of I-gel(*x*)/PB-Sub and PB-gel(*x*)/I-Sub as functions of the PB or I unit ratios in the hydrogel. *S* of I-gel(*x*)/PB-Sub and PB-gel(*x*)/I-Sub increases as the feed ratio of phenylboronic acid and iodophenyl units increases in the hydrogels. Combinations of I-gel(20)/PB-Sub and PB-gel(20)/I-Sub exhibit bonding strengths as high as 8 to 10 kPa. These results suggest that the bonding strengths are closely related to the numbers of functional groups available at the interface as well as the Suzuki-Miyaura coupling reaction at the contact interface.

Finally, we demonstrate selective bonding of hydrogels on glass substrates by placing the I-gels and the AAm-gels at certain positions on PB-Sub (Figure 5c). We depicted the number “12” using the I-gels. After the catalytic reaction with Pd(OAc)_2_, gels on the PB-Sub were rinsed in water. Although the AAm-gels are removed from the PB-Sub, I-gel strongly bond to the PB-Sub, and the number “12” emerges (Movie S2). This demonstration also shows that the I-gel selectively bonds to the PB-Sub through the Suzuki-Miyaura coupling reaction.

## Discussion

We report direct bonding between macroscopic objects through the Suzuki-Miyaura cross-coupling reaction. The adhesion described in this paper is achieved without changing the physical states of the two adherents. Utilizing a certain level of mobility of the functional groups in the gels, covalent bonds are efficiently formed at the surface between gel-gel and gel-glass. PB- and I-gels (or Subs) bond strongly through the Pd-catalyzed reaction, while gels (or Subs) without nucleophilic or electrophilic groups do not. Furthermore, adhesion in this system only occurs between specific surfaces under certain conditions (i.e., in the presence of a Pd catalyst). Another merit is that adhesion is based on covalent bonding, where the bonding strength depends on the number of biphenyl groups formed at the interface by the Suzuki-Miyaura coupling reaction, which can be experimentally controlled by the mol% of the functional groups in the gels, reaction time, etc.

## Methods

### Materials

Acrylamide (AAm), dimethyl sulfoxide (DMSO), and D_2_O were purchased from Wako Pure Chemical Industries, Ltd. Acrylic acid (AA), 2,2*′*-azobis(isobutyronitrile) (AIBN), triethylamine (Et_3_N), potassium carbonate and *N,N′*-methylenebis (acrylamide) (MBAAm) were obtained from Nacalai Tesque Inc. Palladium (II) acetate (Pd(OAc)_2_), (3-aminopropyl)triethoxysilane, 1*H*-benzotriazol-1-yloxytris(dimethylamino)phosphonium hexafluorophosphate (BOP reagent), 4-iodoaniline, 4-iodobenzoic acid, and 4-vinylphenylboronic acid were purchased from Tokyo Chemical Industry Co., Ltd. DMSO-*d_6_* was obtained from Merck & Co., Inc. The glass, No. S1126, which was made from Inorganic Glass (B270) of SCHOTT AG, Plant Grünenplan, was purchased from Matsunami Glass Ind., Ltd. Water, which was purified with a Millipore Elix 5 system, was used to prepare the aqueous solutions. Other reagents were used without further purification.

### Measurements

The ^1^H NMR spectra were recorded at 500 MHz with a JEOL JNM-ECA 500 NMR spectrometer. The FGMAS NMR spectra were recorded at 400 MHz with a JEOL JNM-ECA 400 NMR spectrometer. The sample spinning rate was 8 kHz. In all NMR measurements, chemical shifts were referenced to the solvent values (*δ* = 2.49 ppm and 4.79 ppm for DMSO-*d_6_* and D_2_O, respectively). The contact angles were measured by a Dynamic Contact Angle Analyzer (DCA-700, Kyowa Interface Science Ltd.). The IR spectra of the gels were measured using a JASCO FT/IR-410 spectrometer with a KBr disc, while those of the substrates were measured using a JASCO FT/IR-6000 spectrometer via the attenuated total reflection method (ATR). The mechanical properties of the gels were measured by a mechanical tension testing system (Rheoner, RE-33005, Yamaden Ltd.), while the dynamic viscoelasticity was measured using an Anton Paar MCR301 rheometer. X-ray photoelectron spectroscopy (XPS) data were collected with an AXIS 165 (KRATOS ANALYTICAL) using a monochromatic Al-Kα X-ray source.

## Author Contributions

T.S. performed syntheses, characterizations, and spectroscopic studies. T.K., T.N., Y.K., and Y.T. contributed to the discussion. A.H. and Y.T. conceived and directed the study, contributed to all experiments, and wrote the paper. A.H. oversaw the project as well as helped execute and interpret the results.

## Supplementary Material

Supplementary Information Movie S1

Supplementary Information Movie S2

Supplementary Information Supplementary information
