# Supplementary material for: A Macroscopic Reaction: Direct Covalent Bond Formation between Materials Using a Suzuki-Miyaura Cross-Coupling Reaction
Source: Sci Rep. 2014 Sep 18;4:6348. doi: 10.1038/srep06348 (PMC4166947; doi:10.1038/srep06348)
Supplement: Supplementary Information [file srep06348-s3.pdf]

# - Supplementary Information -

A Macroscopic Reaction: Direct Covalent Bond Formation between Materials Using a Suzuki-Miyaura Cross-Coupling Reaction

*Tomoko Sekine, Takahiro Kakuta, Takashi Nakamura, Yuichiro Kobayashi, Yoshinori Takashima, Akira Harada**

Department of Macromolecular Science, Graduate School of Science, Osaka University, Toyonaka, Osaka 560-0043, Japan.

harada@chem.sci.osaka-u.ac.jp

*** Corresponding author. Telephone +81-6-6850-5445; Fax +81-6-6850-5445**

Page contents

1.	Preparation of PB-gel(*x*)	S3
2.	Preparation of I-gel(*x*)	S4
3.	Characterization of gels (Solid-state ^{1}H FGMAS NMR, FT-IR spectra)	S5-S7
4.	Rupture strength of PB-gel(x)/I-gel(x) as a function of molar ratio of functional groups	S8
5.	Dependency of rupture strength of PB-gel(10)/I-gel(10) on reaction time	S9
6.	Preparation of PB-copolymer	S10
7.	Coupling reaction of PB-copolymer and 4-iodobenzoic acid.	S11
8.	Suzuki-Miyaura cross-coupling reaction between 4-iodoaniline and PB-copolymer.	S12
9.	Preparation of I-copolymer	S13
10.	Coupling reaction between PB-copolymer and I-copolymer	S14
11.	Preparation of PB-Sub and I-Sub	S15
12.	Characterization of PB-Sub and I-Sub (Contact angles, FT-IR ATR, X-ray photoelectron spectroscopy)	S16-S18

1. Preparation of PB-gel(*x*)

Typical procedure for PB-gel(10). AAm (183 mg, 2.6 mmol), 4-vinylphenylboronic acid (PB) (44 mg, 0.30 mmol), MBAAm (19 mg, 0.12 mmol) and AIBN (2.5 mg, 0.015 mmol) were dissolved in DMSO (1.5 mL). The solution was purged with argon gas for 1 h and was then heated at 70 °C overnight to form a gel. The gel was washed repeatedly with DMSO, followed by water, respectively. The gel was soaked in water and used as hydrogels.

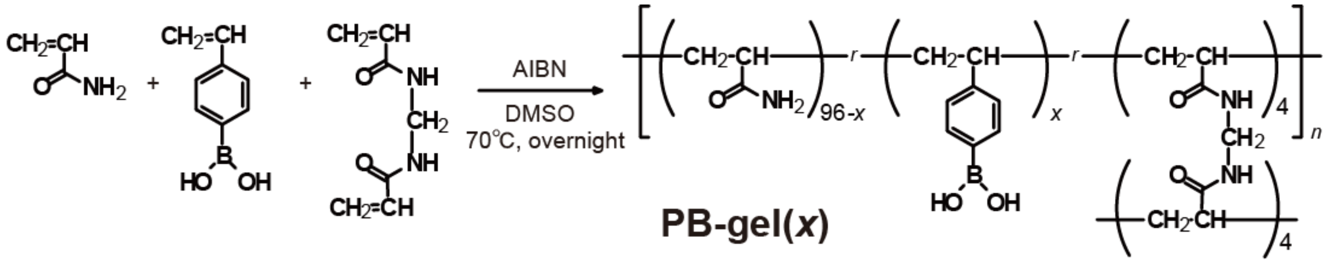

Scheme S1. Preparation of PB-gel(*x*).

Table S1. Preparation of PB-gel(*x*) varying the mol% of the PB unit.

PB-gel(*x*)	PB		AAm		MBAAm	AIBN
	/ mmol	/mol%	/ mmol	/mol%	/ mmol	/ mmol
PB-gel(2)	0.06	2	2.8	94	0.12	0.015
PB-gel(5)	0.15	5	2.7	91	0.12	0.015
PB-gel(10)	0.30	10	2.6	86	0.12	0.015
PB-gel(15)	0.45	15	2.4	81	0.12	0.015
PB-gel(20)	0.60	20	2.3	76	0.12	0.015

2. Preparation of I-gel(*x*)

Typical procedure for I-gel(10). AAm (188 mg, 2.6 mmol), AA (20.6 μL, 0.30 mmol), MBAAm (19 mg, 0.12 mmol) and AIBN (2.5 mg, 0.015 mmol) were dissolved in DMSO (1.5 mL). The solution was purged with argon gas for 1 h and was then heated at 70 °C overnight to form a gel. The gel was soaked in 4 mL DMSO solution containing 4-iodoaniline (I) (197 mg, 0.90 mmol), BOP reagent (400 mg, 0.90 mmol) and Et_3N (124 μL, 0.90 mmol) and shaken for 48 hours at 25 °C. After the reaction, the gel was washed repeatedly with DMSO, followed by water, respectively. The gel was soaked in water and used as hydrogels.

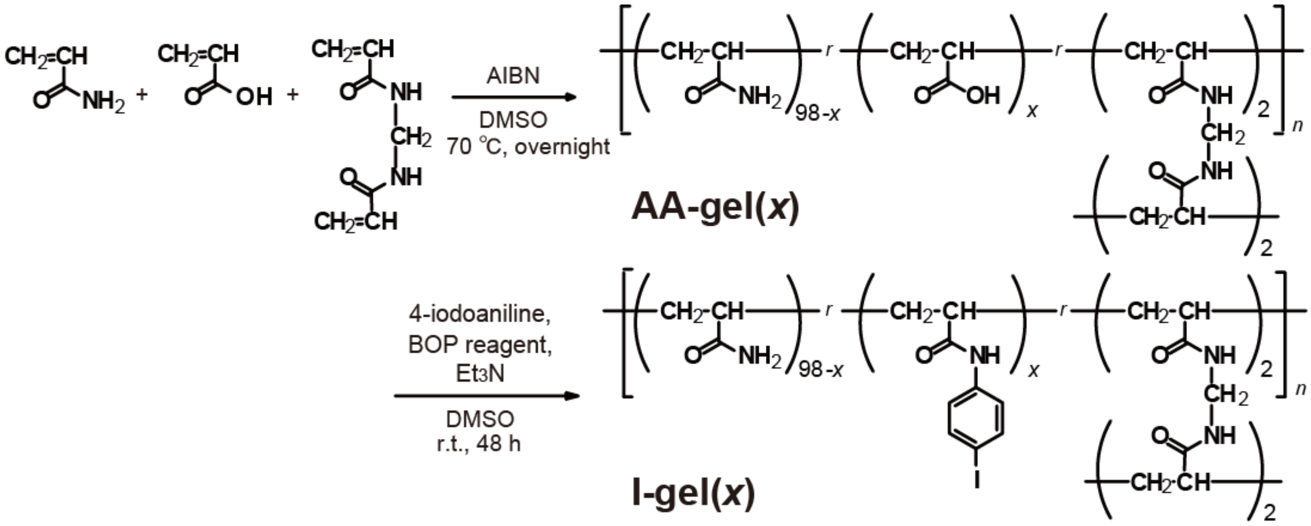

Scheme S2. Preparation of I-gel(*x*).

Table S2. Preparation of AA-gel(*x*) varying the mol% of the AA unit.

AA-gel(*x*)	AA		AAm		MBAAm	AIBN
	/ mmol	/mol%	/ mmol	/mol%	/ mmol	/ mmol
AA-gel(2)	0.06	2	2.9	96	0.06	0.015
AA-gel(5)	0.15	5	2.8	93	0.06	0.015
AA-gel(10)	0.30	10	2.6	88	0.06	0.015
AA-gel(15)	0.45	15	2.5	83	0.06	0.015
AA-gel(20)	0.60	20	2.3	78	0.06	0.015

3. Characterization of gels (Solid-state ^{1}H FGMAS NMR, FT-IR spectra)

Solid-state ^{1}H FGMAS NMR

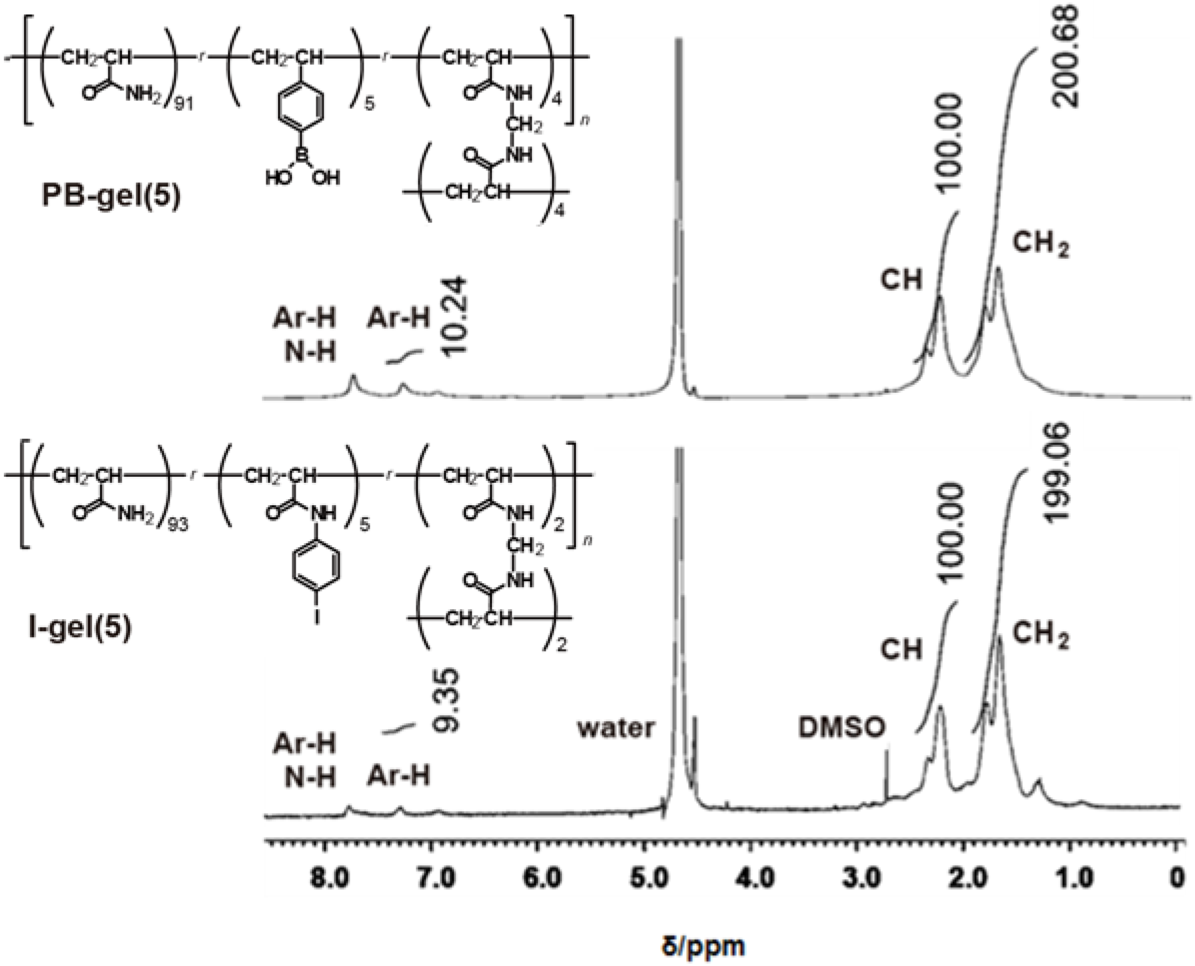

Figure S1. ^{1}H solid-state field gradient magic angle spinning nuclear magnetic resonance (^{1}H FGMAS NMR) spectra of PB-gel(5) and I-gel(5), respectively. (Immersed in D_2O, 400 MHz, 30 °C, rotation frequency = 8 kHz).

FT-IR spectra

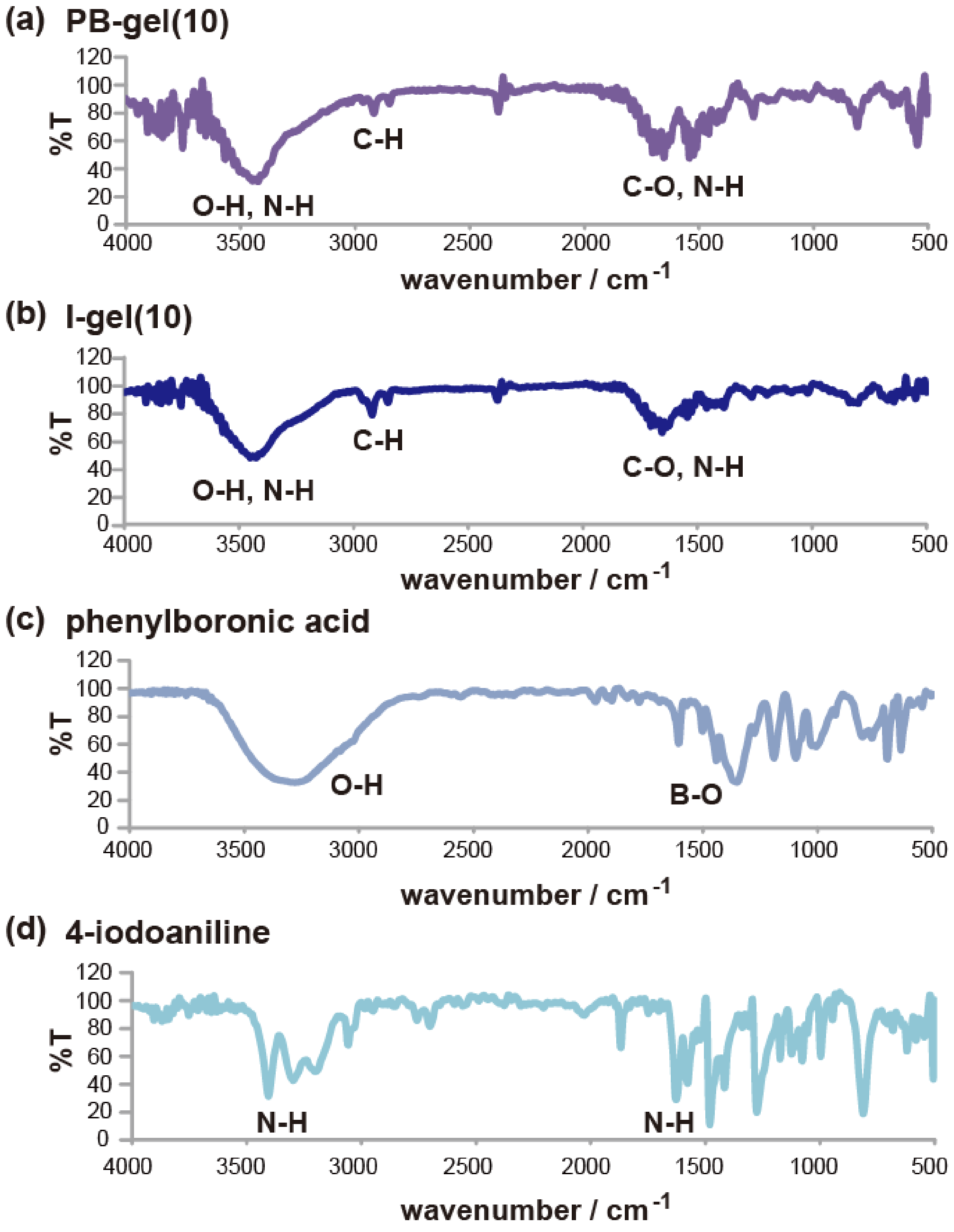

Figure S2. FT-IR spectra of (a) PB-gel(10), (b) I-gel(10), (c) phenylboronic acid, and (d) 4-iodoaniline, respectively. %T represents transmittance percentage. (KBr disc, r.t.)

PB-gel(10): 3378, 3218 cm^{-1} (O-H stretching vibration, N-H asymmetric stretching vibration, and N-H symmetric stretching vibration), 2865, 2790 cm^{-1} (methylene C-H stretching vibration) 1654 cm^{-1} (C=O stretching vibration, N-H bending vibration).

I-gel(10): 3386, 3201 cm^{-1} (O-H stretching vibration, N-H asymmetric stretching vibration, and N-H symmetric stretching vibration), 2898, 2790 cm^{-1} (methylene C-H stretching vibration) 1655 cm^{-1} (C=O stretching vibration, N-H bending vibration).

Table S3. The mol% of functional group in PB-gels calculated by integral values of ^{1}H FGMAS NMR signals.

	PB unit / mol%
PB-gel(2)	3.5
PB-gel(5)	5.1
PB-gel(10)	7.5
PB-gel(15)	12.3
PB-gel(20)	25.1

Table S4. The mol% of functional group in I-gels calculated by integral values of ^{1}H FGMAS NMR signals.

	I unit / mol%
I-gel(2)	3.2
I-gel(5)	4.7
I-gel(10)	6.1
I-gel(15)	9.0
I-gel(20)	14.1

4. Rupture strengths of PB-gel(*x*)/I-gel(*x*) as a function of molar ratios of functional groups

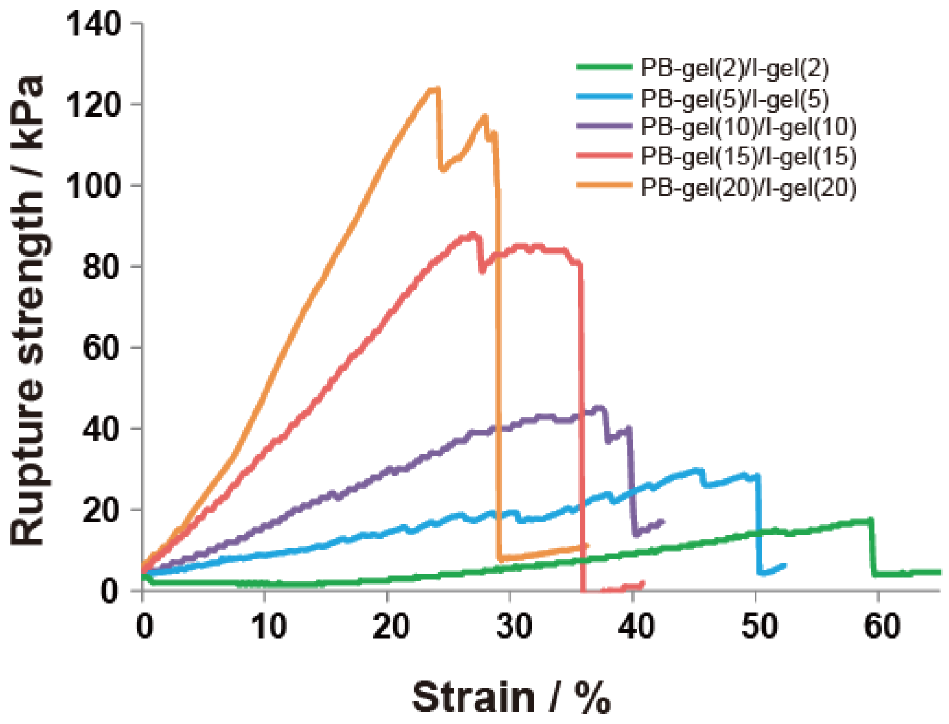

Figure S3. Stress-strain curves of PB-gel(*x*)/I-gel(*x*) (*x*; 2, 5, 10, 15, and 20).

5. Dependency of rupture strength of PB-gel(10)/I-gel(10) on reaction time

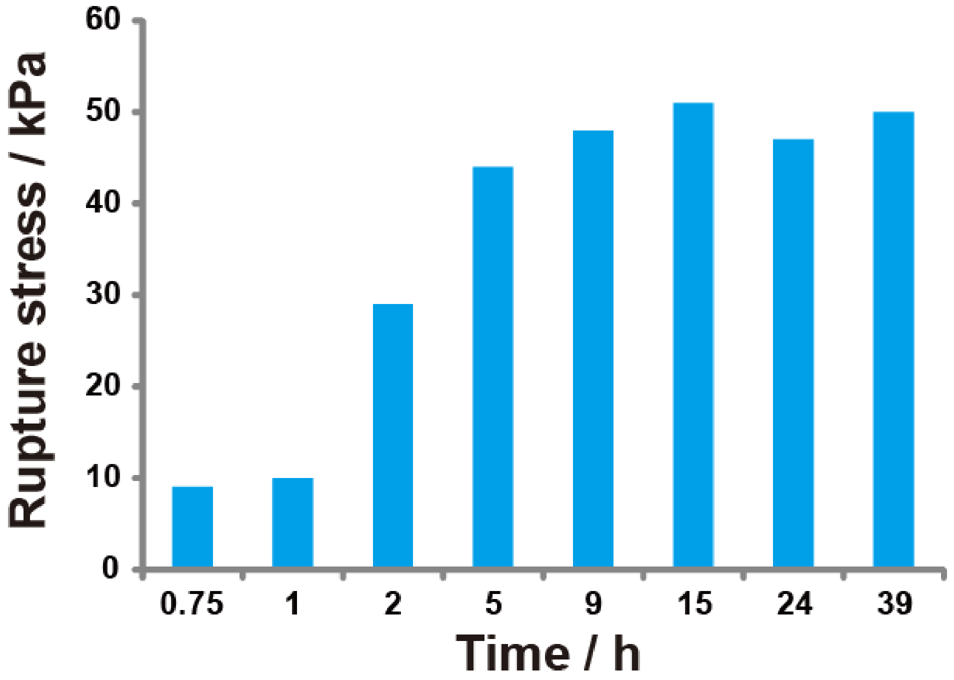

Figure S4. Dependency of rupture stress values of PB-gel(10)/I-gel(10) on reaction time of Suzuki-Miyaura cross-coupling. See the main text for reaction conditions.

6. Preparation of PB-copolymer

Typical procedure for PB-copolymer. AAm (511 mg, 7.2 mmol), 4-vinylphenylboronic acid (118 mg, 0.80 mmol) and AIBN (13.5 mg, 0.08 mmol) were dissolved in DMSO (5 mL). The solution was purged with argon gas for 1 h and was then heated at 70 °C overnight. The solution was poured into methanol and the white precipitate was obtained by filtration, followed by washing with methanol and drying in vacuo. at 50 °C. Yield 76%, 564 mg.

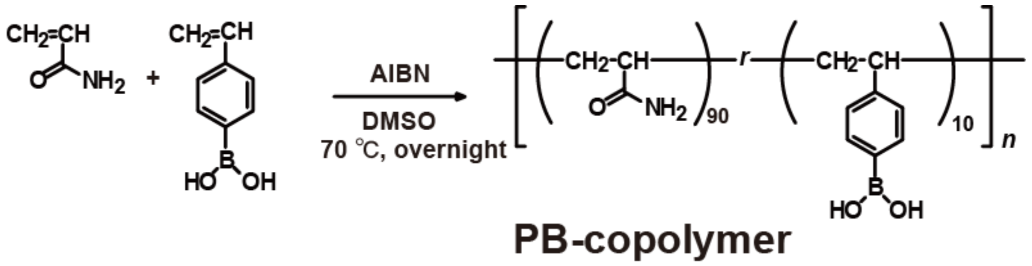

Scheme S3. Preparation of PB-copolymer. *x* represents the mol% of functional groups (4-phenylboronic acid).

7. Coupling reaction of PB-copolymer and 4-iodobenzoic acid

PB-copolymer (80 mg, 8.9 mmol of PB units) and 4-iodobenzoic acid (30 mg, 12 mmol) were dissolved in 45 mL of an aqueous solution of K_2CO_3 (31 mmol). 0.10 mL of acetone solution of $Pd(OAc)_2$ (0.05 mmol) was added and stirred for 24 hours. The solution was poured into methanol and the white precipitate was obtained by filtration, followed by washing with methanol and drying in vacuo. at 50 °C. Yield 58%, 56mg.

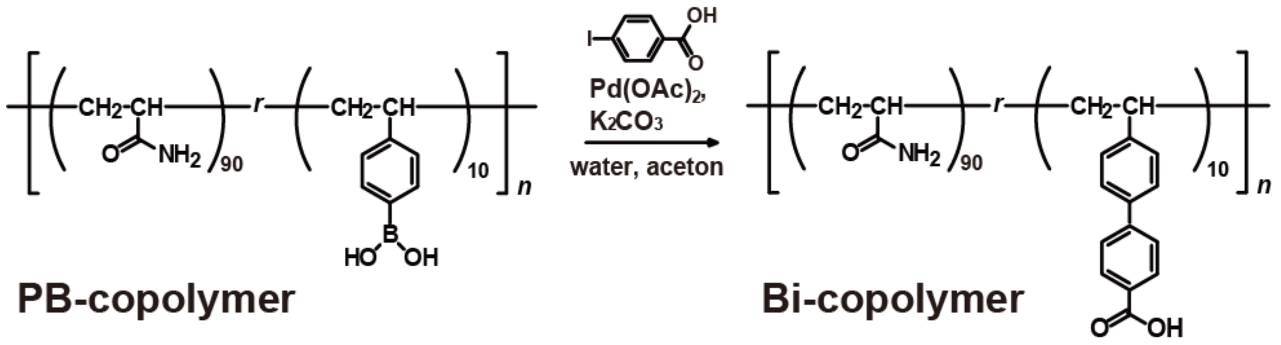

Scheme S4. Coupling reaction of PB-copolymer and 4-iodobenzoic acid. *x* represents the mol% of functional groups of PB-copolymer (PB group) and biphenyl carboxylic acid moiety of Bi-copolymer.

8. Suzuki-Miyaura cross-coupling reaction between 4-iodoaniline and PB-copolymer.

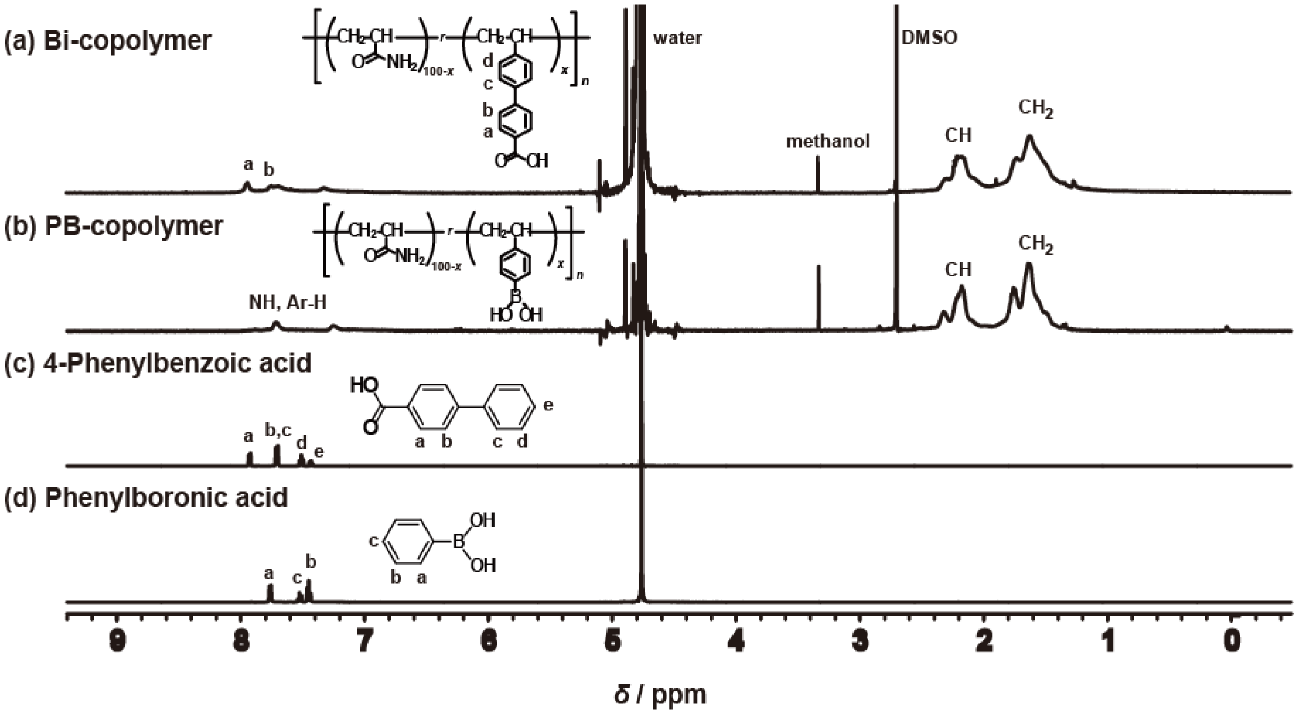

Figure S5. ^{1}H NMR spectra of the biphenyl copolymer (Bi-copolymer) formed by Suzuki-Miyaura coupling reaction, and those of related compounds (500 MHz, D_2O, 30 °C). (a) Bi-copolymer, the product of the coupling reaction between PB-copolymer and 4-iodobenzoic acid. (b), PB-copolymer. (c) 4-Phenylbenzoic acid. (d) Phenylboronic acid .

9. Preparation of I-copolymer

Typical procedure for I-copolymer: AAm (550 mg, 7.7 mmol), AA (16 μL, 0.24 mmol) and AIBN (6.5 mg 0.04 mmol) were dissolved in DMSO (4 mL). The solution was purged with argon gas for 1 h and was then heated at 70 °C overnight. The white precipitate was obtained by reprecipitation of the solution with methanol and filtration. The precipitate was dissolved in 4 mL DMSO containing 4-iodoaniline (197 mg, 0.90 mmol), BOP reagent (400 mg, 0.90 mmol) and Et_3N (124 μL, 0.90 mmol). After the solution was stirred overnight at room temperature, the solution was poured into methanol and the white precipitate was obtained by filtration, followed by washing with methanol and drying in vacuo. at 50 °C. Yield 69%, 430 mg.

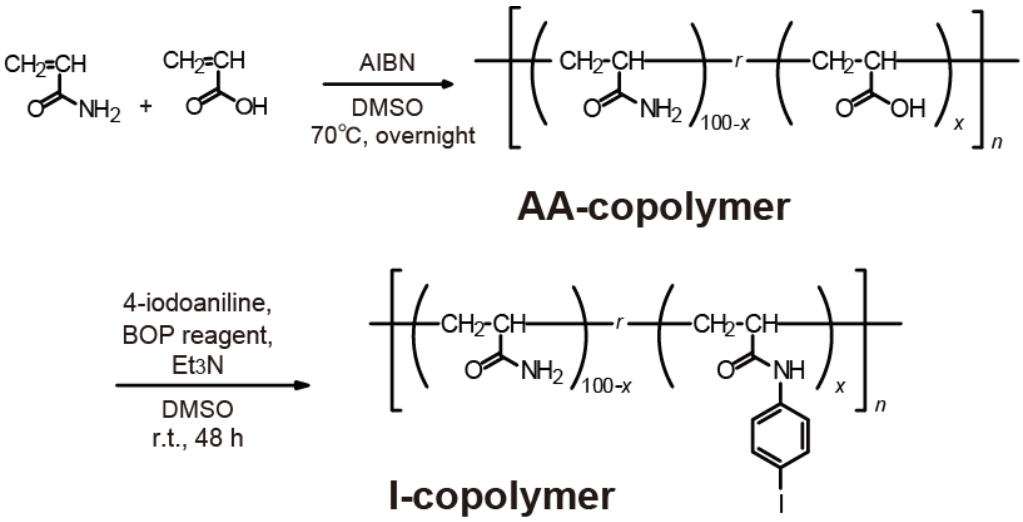

Scheme S5. Preparation of I-copolymer. *x* represents the mol% of AA unit in AA-copolymer and I group in I-copolymer.

10. Coupling reaction between PB-copolymer and I-copolymer

Typical procedure for the coupling reaction: PB-copolymer (6 mg, 6.6 μmol of PB units) and I-copolymer (15 mg, 4.5 μmol of I units) were dissolved in 140 μL of aqueous solution of K_2CO_3 (0.10 mmol). 0.01 mL of acetone solution of $Pd(OAc)_2$ (5 μmol) was added and stirred at 25 °C for 3 hours, after which the sample was directly used for rheological measurements.

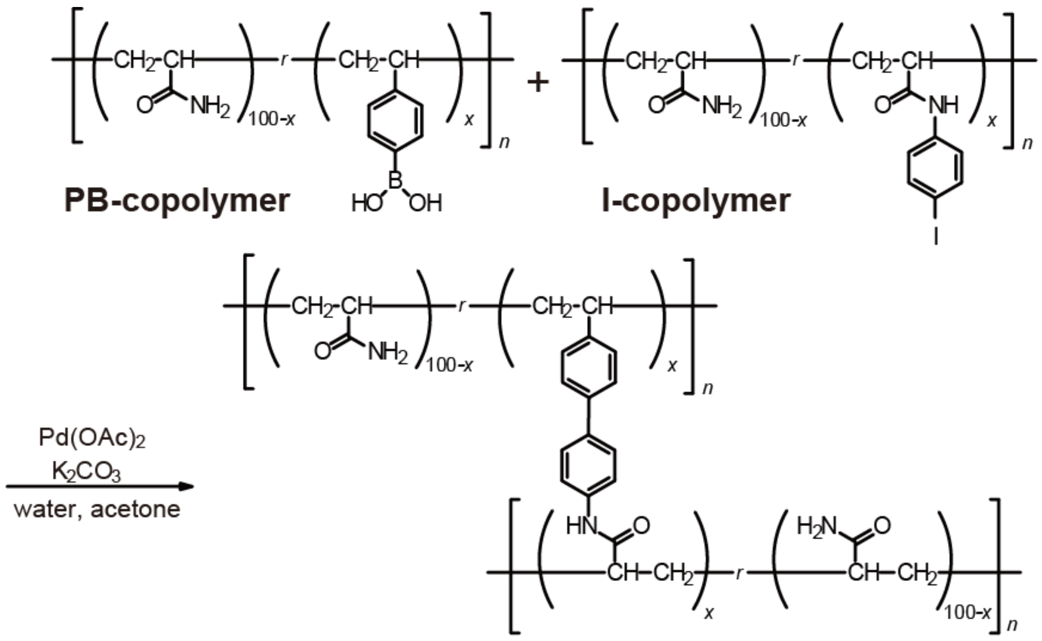

Scheme S6. Coupling reaction between PB- and I-copolymers. *x* represents the mol% of functional groups (PB group or I group).

Table S5. Dynamic viscoelastic properties of the product of Suzuki-Miyaura cross-coupling reactions (entry 1) and its controls (entries 2-4).

Entry	PB-copolymer /mg	I-copolymer /mg	K_2CO_3 /mg	$Pd(OAc)_2$ /mg	G' /Pa	G'' /Pa	State
1	6	15	40	1	3.6×10^{2}	1.5×10^{1}	gel
2	6	15	40	–	6.1×10^{-1}	4.1×10^{-1}	sol
3	6	–	20	–	5.5×10^{-2}	3.0×10^{-2}	sol
4	–	15	20	–	1.5×10^{-1}	1.2×10^{-1}	sol

*The frequency range ; 1 rad•s^{-1}

11. Preparation of PB-Sub and I-Sub

Glass plates (MATSUNAMI No. S1126, 76 mm × 26 mm ×1.0 mm) were cut into 26 mm×26 mm ×1.0 mm pieces. 8 plates of the glass were ultrasonicated in toluene, acetone, and methanol for 15 min, respectively, and subsequently dried. The substrates were cleaned with a UV–O_3 cleaner (Filgen UV253S) for 12 hours to create a hydrophilic surface. The cleaned substrates were immersed in a toluene (70 mL) solution with 3-aminopropyl triethoxy silane (APTES) (1.6 mL, 6.8 mmol) at room temperature and shaken overnight. These substrates were washed four times with toluene, and immersed in a DMSO (70 mL) solution with 4-iodobenzoic acid (870 mg, 3.5 mmol), BOP reagent (1.86 g, 4.2 mmol) and Et_3N (0.6 mL, 4.3 mmol) for two days at room temperature. Each substrate was washed with DMSO and water repeatedly.

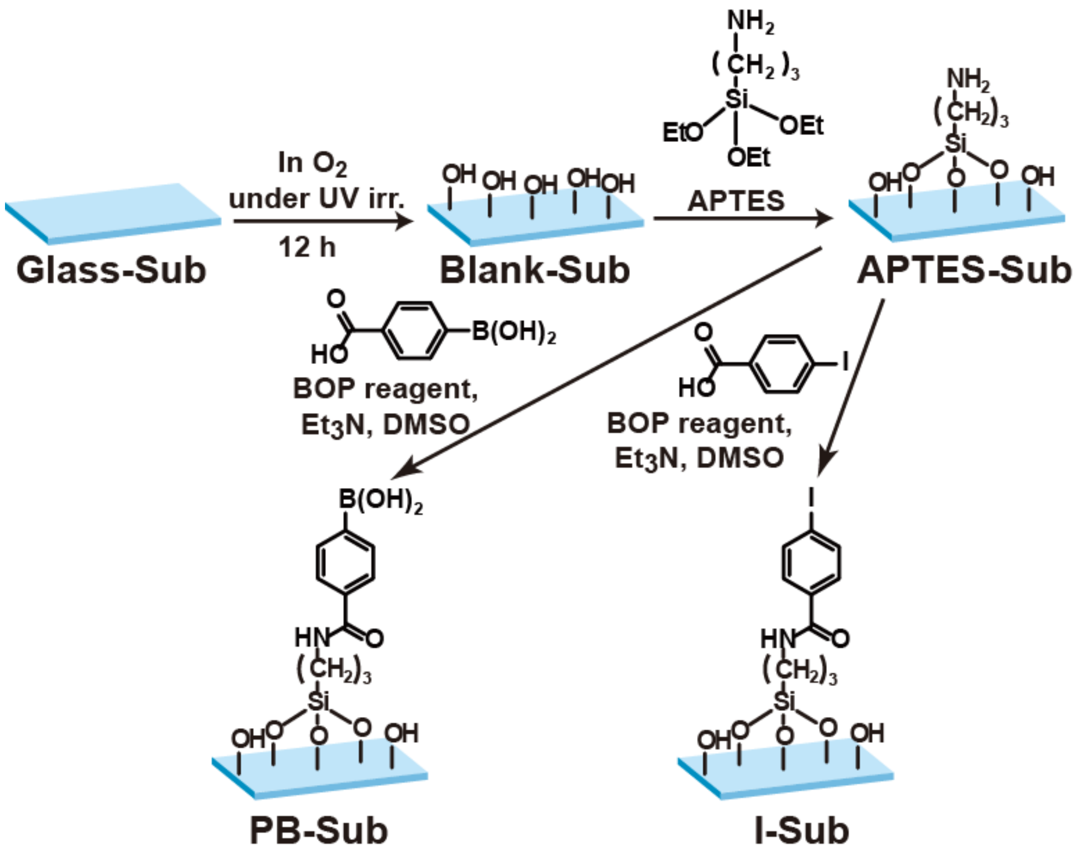

Scheme S7. Preparation of PB-Sub and I-Sub.

12. Characterization of PB-Sub and I-Sub (Contact angles, FT-IR ATR method, X-ray photoelectron spectroscopy)

Contact angles.

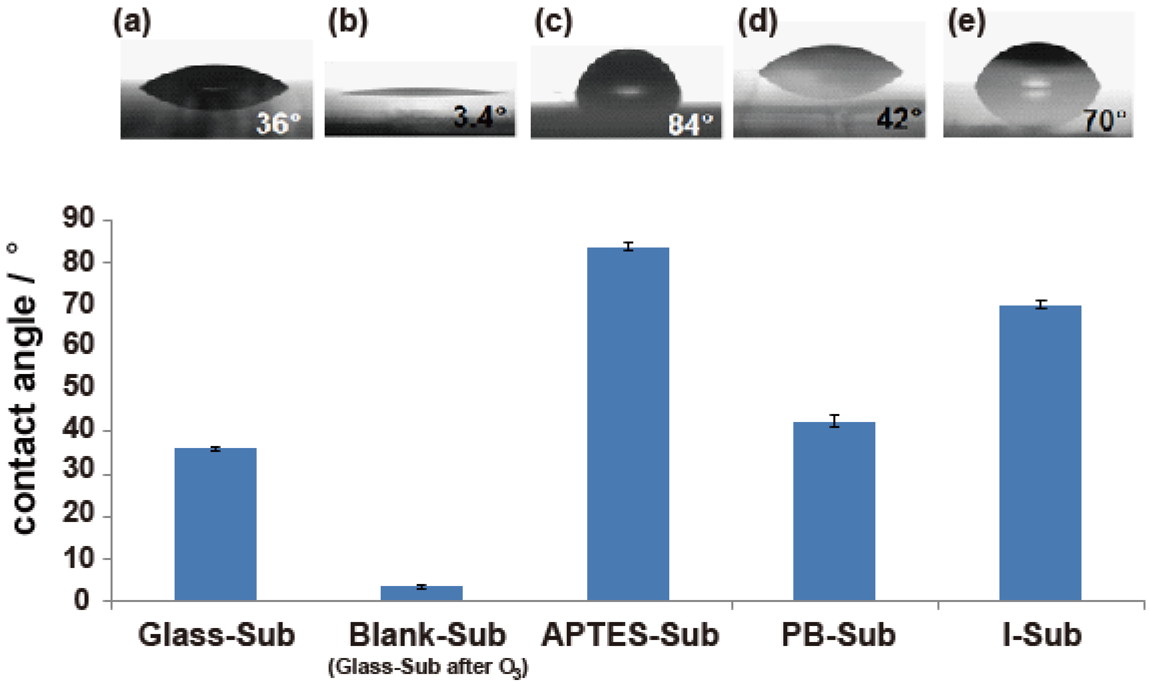

Figure S6. Pictures of water droplets on the surfaces and contact angle of Glass-Sub (a), Blank-Sub after treating Glass-Sub with O_3 (b), APTES-Sub (c), PB-Sub (d), and I-Sub (e). The error bars were standard deviations of 3 samples.

FT-IR ATR method

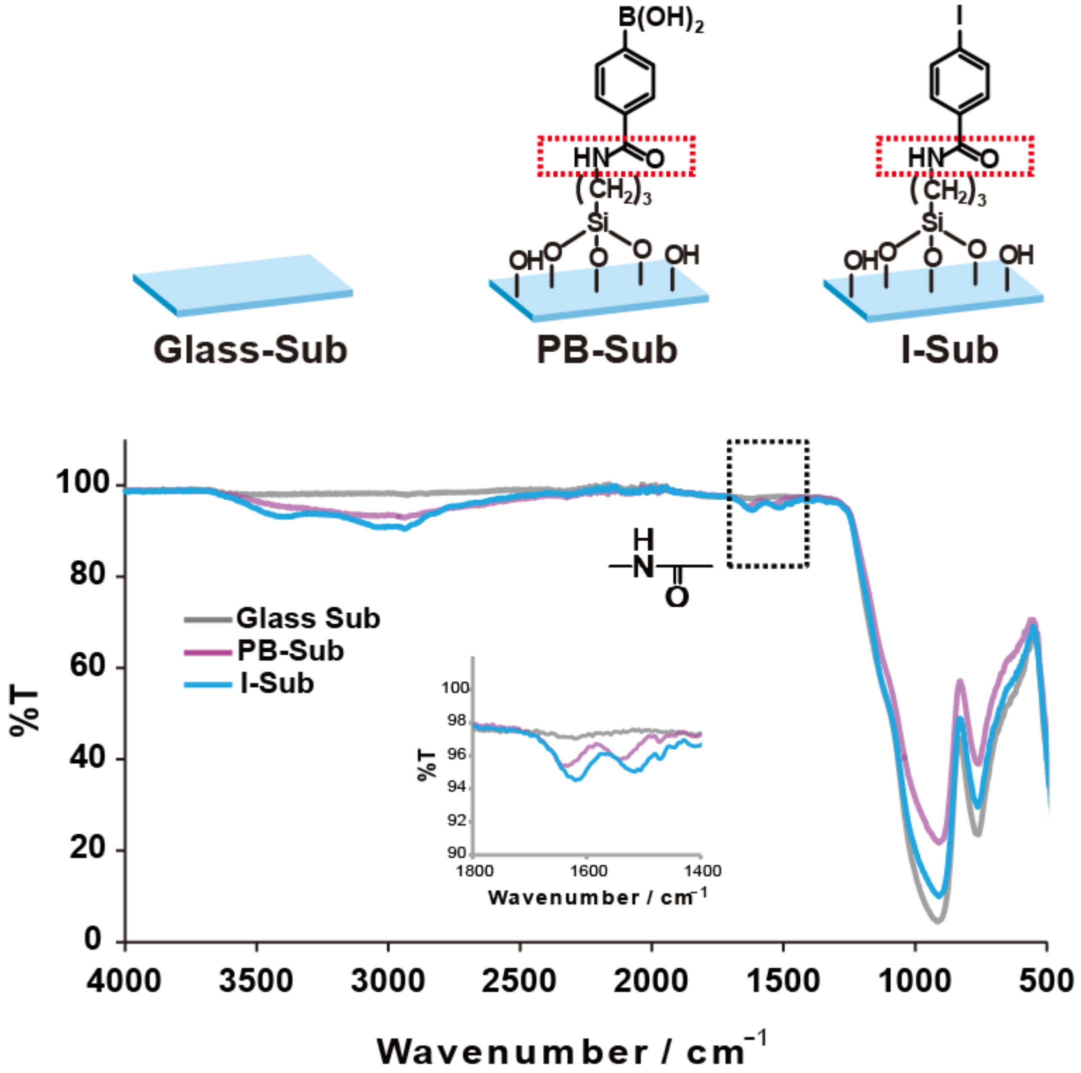

Figure S7. FT-IR spectra of Glass-Sub (gray), PB-Sub (red), and I-Sub (blue). %T represents transmittance percentage.

X-ray photoelectron spectroscopy (XPS)

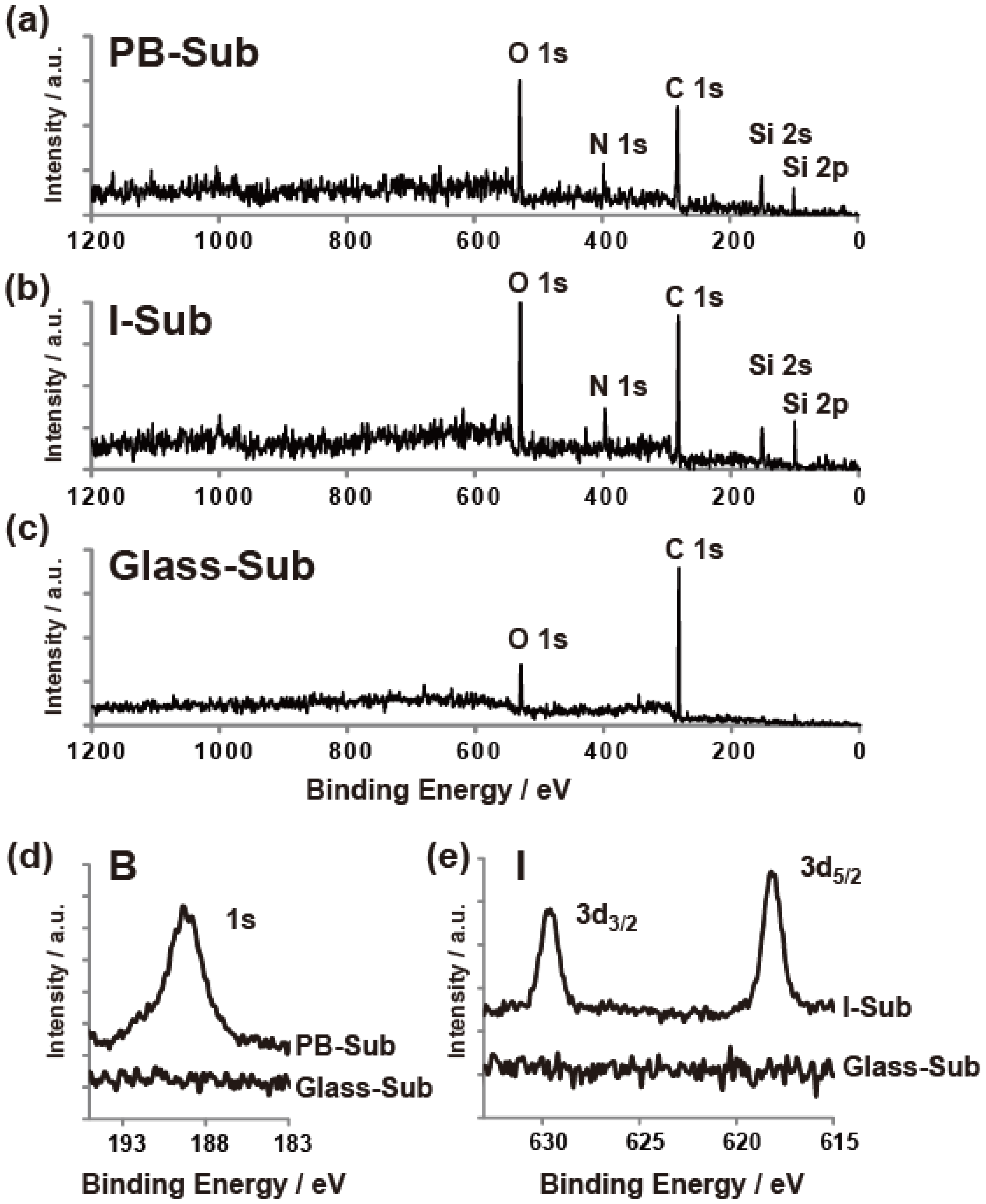

Figure S8. X-ray photoelectron spectroscopy (XPS) spectra of PB-Sub (a), I-Sub (b), Glass-Sub (c). (d) and (e) show the high-resolution spectra focused on the PB-Sub (d) and the I-Sub (e).

Figure legends

Figure S1. ^{1}H solid-state field gradient magic angle spinning nuclear magnetic resonance (^{1}H FGMAS NMR) spectra of PB-gel(5) and I-gel(5), respectively. (Immersed in D_2O, 400 MHz, 30 °C, rotation frequency = 8 kHz).

Figure S2. FT-IR spectra of (a) PB-gel(10), (b) I-gel(10), (c) phenylboronic acid, and (d) 4-iodoaniline, respectively. %T represents transmittance percentage. (KBr disc, r.t.)

Figure S3. Stress-strain curves of PB-gel(*x*)/I-gel(*x*) (*x*; 2, 5, 10, 15, and 20).

Figure S4. Dependency of rupture stress values of PB-gel(10)/I-gel(10) on reaction time of Suzuki-Miyaura cross-coupling. See the main text for reaction conditions.

Figure S5. ^{1}H NMR spectra of the biphenyl copolymer (Bi-copolymer) formed by Suzuki-Miyaura coupling reaction, and those of related compounds (500 MHz, D_2O, 30 °C). (a) Bi-copolymer, the product of the coupling reaction between PB-copolymer and 4-iodobenzoic acid. (b), PB-copolymer. (c) 4-Phenylbenzoic acid. (d) Phenylboronic acid .

Figure S6. Pictures of water droplets on the surfaces and contact angle of Glass-Sub (a), Blank-Sub after treating Glass-Sub with O_3 (b), APTES-Sub (c), PB-Sub (d), and I-Sub (e). The error bars were standard deviations of 3 samples.

Figure S7. FT-IR spectra of Glass-Sub (gray), PB-Sub (red), and I-Sub (blue). %T represents transmittance percentage.

Figure S8. X-ray photoelectron spectroscopy (XPS) spectra of PB-Sub (a), I-Sub (b), Glass-Sub (c). (d) and (e) show the high-resolution spectra focused on the PB-Sub (d) and the I-Sub (e).

Scheme S1. Preparation of PB-gel(*x*).

Scheme S2. Preparation of I-gel(*x*).

Scheme S3. Preparation of PB-copolymer. *x* represents the mol% of functional groups (4-phenylboronic acid).

Scheme S4. Coupling reaction of PB-copolymer and 4-iodobenzoic acid. *x* represents the mol% of functional groups of PB-copolymer (PB group) and biphenyl carboxylic acid moiety of Bi-copolymer.

Scheme S5. Preparation of I-copolymer. *x* represents the mol% of AA unit in AA-copolymer and I group in I-copolymer.

Scheme S6. Coupling reaction between PB- and I-copolymers. *x* represents the mol% of functional groups (PB group or I group).

Scheme S7. Preparation of PB-Sub and I-Sub.

Table S1. Preparation of PB-gel(*x*) varying the mol% of the PB unit.

Table S2. Preparation of AA-gel(*x*) varying the mol% of the AA unit.

Table S3. The mol% of functional group in PB-gels calculated by integral values of ^{1}H FGMAS NMR signals.

Table S4. The mol% of functional group in I-gels calculated by integral values of ^{1}H FGMAS NMR signals.

Table S5. Dynamic viscoelastic properties of the product of Suzuki-Miyaura cross-coupling reactions (entry 1) and its controls (entries 2-4).
